# Hepatic insulin resistance both in prediabetic and diabetic patients determines postprandial lipoprotein metabolism: from the CORDIOPREV study

**DOI:** 10.1186/s12933-016-0380-y

**Published:** 2016-04-19

**Authors:** A. Leon-Acuña, J. F. Alcala-Diaz, J. Delgado-Lista, J. D. Torres-Peña, J. Lopez-Moreno, A. Camargo, A. Garcia-Rios, C. Marin, F. Gomez-Delgado, J. Caballero, B. Van-Ommen, M. M. Malagon, P. Perez-Martinez, J. Lopez-Miranda

**Affiliations:** Lipid and Atherosclerosis Unit, IMIBIC/Reina Sofia University Hospital/University of Cordoba, Cordoba, Spain; CIBER Fisiopatologia Obesidad y Nutricion (CIBEROBN), Instituto de Salud Carlos III, Madrid, Spain; Biochemical Laboratory, Hospital Universitario Reina Sofia, Cordoba, Spain; TNO, Zeist, The Netherlands; Department of Cell Biology, Physiology, and Immunology, IMIBIC/Reina Sofia University Hospital/University of Cordoba, Cordoba, Spain

**Keywords:** Phenotypic flexibility, Triglycerides, Postprandial lipemia, Prediabetic, CORDIOPREV study, Insulin resistance

## Abstract

**Background/aims:**

Previous evidences have shown the presence of a prolonged and exaggerated postprandial response in type 2 diabetes mellitus (T2DM) and its relation with an increase of cardiovascular risk. However, the response in prediabetes population has not been established. The objective was to analyze the degree of postprandial lipemia response in the CORDIOPREV clinical trial (NCT00924937) according to the diabetic status.

**Methods:**

1002 patients were submitted to an oral fat load test meal (OFTT) with 0.7 g fat/kg body weight [12 % saturated fatty acids (SFA), 10 % polyunsaturated fatty acids (PUFA), 43 % monounsaturated fatty acids (MUFA), 10 % protein and 25 % carbohydrates]. Serial blood test analyzing lipid fractions were drawn at 0, 1, 2, 3 and 4 h during postprandial state. Postprandial triglycerides (TG) concentration at any point >2.5 mmol/L (220 mg/dL) has been established as undesirable response. We explored the dynamic response in 57 non-diabetic, 364 prediabetic and 581 type 2 diabetic patients. Additionally, the postprandial response was evaluated according to basal insulin resistance subgroups in patients non-diabetic and diabetic without pharmacological treatment (N = 642).

**Results:**

Prevalence of undesirable postprandial TG was 35 % in non-diabetic, 48 % in prediabetic and 59 % in diabetic subgroup, respectively (p < 0.001). Interestingly, prediabetic patients displayed higher plasma TG and large triacylglycerol-rich lipoproteins (TRLs-TG) postprandial response compared with those non-diabetic patients (p < 0.001 and p = 0.003 respectively). Moreover, the area under the curve (AUC) of TG and AUC of TRLs-TG was greater in the prediabetic group compared with non-diabetic patients (p < 0.001 and p < 0.005 respectively). Patients with liver insulin resistance (liver-IR) showed higher postprandial response of TG compared with those patients with muscle-IR or without any insulin-resistance respectively (p < 0.001).

**Conclusions:**

Our findings demonstrate that prediabetic patients show a lower phenotypic flexibility after external aggression, such as OFTT compared with nondiabetic patients. The postprandial response increases progressively according to non-diabetic, prediabetic and type 2 diabetic state and it is higher in patients with liver insulin-resistance. To identify this subgroup of patients is important to treat more intensively in order to avoid future cardiometabolic complications.

## Background

Prediabetes status forms an intermediate stage in the natural history of type 2 diabetes mellitus (T2DM), and behaves a high risk of cardiovascular complications. It is estimated that 10 % of population have prediabetes, although only one third know it. The average risk of development diabetes increases 0.7 % per year in people with normal glucose levels, and 5–10 % per year in prediabetic patients [1]. Interestingly, at this stage of the disease, it is possible to return a normal state [2]. T2DM has been associated with abnormal postprandial lipoprotein metabolism, with a significant delay in the clearance of lipoproteins, including triglycerides (TG) and chylomicrons [3]. This fact could support the hypothesis to consider T2DM as a systems disease with loss of flexibility in one or more metabolic processes involved. Therefore, the capacity to adapt in time and location to alterations in external factors, such as environmental conditions, is called phenotypic flexibility [4]. One biomarker of this phenotypic inflexibility is the degree of postprandial triglyceride response. In this regard, the oral fat load test is a classic example of a challenge test [5] and the exaggerated response has been related to proatherogenic conditions. Moreover clinical studies provide evidence that exposure to postprandial lipoproteins is associated with cardiovascular diseases [6, 7]. However at this point, little is known about the role of postprandial lipemia in the prediabetes status. In fact, it is important to understand whether the underlying causes of metabolic inflexibility may influence the maintenance of overall triglycerides homoeostasis in the prediabetic status.

Based in this previous evidence, the aim of this study was examined the degree of postprandial lipemia response measured with the fat tolerance test according to their diabetic status: prediabetic, non-diabetic and diabetic patients from the large cohort of CORDIOPREV clinical trial (NCT00924937). In a next step, we explored the postprandial response according to the presence or absence of muscle and/or liver insulin resistance.

## Methods

### Population

The current work was conducted within the framework of the CORDIOPREV study. The CORDIOPREV study is an ongoing prospective, randomized, opened, controlled trial including 1002 patients with coronary heart disease (CHD), which had their last coronary event more than 6 months before of the enrolment in two different dietary models (Mediterranean and low-fat) over a period of 5 years in addition to conventional treatment for coronary heart disease [8].

Patients were recruited from November 2009 to February 2012, mostly at the Reina Sofia University Hospital (Cordoba, Spain), but other centers from the Cordoba and Jaen provinces were also included. Inclusion and exclusion criteria have been reported previously [9]. In summary, patients were eligible if they were older than 20 years, but younger of 75, had established CHD without clinical events in the last 6 months, were thought to follow a long-term dietary intervention and did not have severe diseases or expected life expectancy lower than 5 years. Patients were categorized depending on the presence of prediabetes criteria, T2DM diagnosis or non-diabetes subgroup. Later, non-diabetic and diabetic patients without pharmacological treatment were divided in four groups according the present of muscle and/or liver insulin resistance (IR): liver-IR, muscle-IR, insulin and muscle IR, without liver or muscle-IR.

### Criteria for prediabetes

Patients were classified according to American Diabetes Association (ADA) prediabetes criteria classification:

- Impaired fasting glucose (IFG): 100 mg/dL (5.6 mmol/L) to 125 mg/dL (6.9 mmol/L) and/or
- Impaired glucose tolerance (IGT): 2 h plasma glucose in the 75 gr OGTT 140 mg/dL (7.8 mmol/L) to 199 mg/dL (11 mmol/L) and/or
- Hemoglobin glycated (HbA1c) plasma levels 5.7–6.4 %.

All patients gave written informed consent to participate in the study. The trial protocol and all amendments were approved by the local ethics committees, following the Helsinki declaration and the good clinical practices.

### Study design

Before participants were enrolled in two different dietary models (Mediterranean diet and Low fat diet) from CORDIOPREV study, they received an oral fat tolerance test using a weight-adjusted meal (0.7 g fat and 5 mg cholesterol per kg body weight) with 12 % saturated fatty acids (SFA), 10 % polyunsaturated fatty acids (PUFA), 43 % monounsaturated fatty acids (MUFA), 10 % protein and 25 % carbohydrates (CHO). Meal preparation was performed by a group of nutritionists with olive oil, skimmed milk, white bread, cooked egg yolks and tomatoes.

### Methodology of the oral glucose tolerance test

Patients underwent a standard oral glucose tolerance test (OGTT) at baseline. After an overnight fast, blood was sampled from a vein before oral glucose intake (0-min) and then, after 75 gr flavoured glucose load (Trutol 75; Custom Laboratories, Baltimore, MD), blood samples were taken at 30, 60, 90 and 120 min to determine the glucose and insulin concentrations [10].

### Estimation of insulin resistance, insulin secretion and beta cell function indices

The indices used to determined tissue-specific IR were the validated hepatic insulin resistance index (HIRI) and the muscle insulin sensitibity index (MISI) [11]. HIRI was estimated by fasting insulin (mU/L) × fasting glucose (mg/dL). MISI was measured MISI = (dG/dt)/mean plasma insulin concentration, where dG/dt is the rate of decay of plasma glucose concentration from its peak value to its nadir during the OGTT.

### Determination of muscle and liver insulin resistance groups

At baseline, the patients were distributed into four groups according to the presence or absence of muscle and/or liver IR. For this purpose, we have used a method based on that described by Abdul-Ghani et al. [12]. The patients were divided into tertiles according their HIRI and MISI. The highest tertile of the HIRI and the lowest tertile in MISI were considered to indicate IR in each organ respectively. A second operational definition based on the median value for IR in skeletal muscle and liver resulted in similar results.

### Methodology of the oral fat tolerance test

Previously to the starting of the test, the patients had been fasting for 12 h and were asked to refrain from smoking during the fasting period and from alcohol intake during the preceding 7 days. They were also asked to avoid strenuous physical activity the day before the test given. At 8:00 a.m. patients presented in the laboratory, completed anthropometric (weight, height, waist circumference, BMI, blood pressure) and biochemical measurements, donated a fasting blood sample and under supervision, ingested the fatty food meal. The breakfast was eaten in 20 min. After the meal, volunteers were resting and consumed no food for 5 h, but were allowed to drink water. Blood samples for biochemical testing were collected before the meal and every hour during the next 4 h, following recommendations for an oral fat tolerance test proposed by Mihas et al. in a recent meta-analysis [13]. Postprandial TG concentration at any point >2.5 mmol/L (220 mg/dL) has been established as undesirable response [5].

### Laboratory test

Venous blood was sampled from the antecubital vein and collected into vacutainer tubes with no anticoagulant and to tubes containing EDTA, and immediately transferred to 4 °C. To minimize proteolytic degradation, plasma was supplemented with protease inhibitor cocktail (Roche Diagnostic, Germany) 40 μL per mL of plasma. Plasma and serum samples were frozen at −80 °C for further biochemical analysis. Serum parameters were measured in Architect c-16000 analyzers (Abbott^®^, Chicago, Illinois, USA) by spectrophotometric techniques (enzymatic colorimetric methods): hexokinase method for glucose, and oxidation-peroxidation for total cholesterol, HDL-C and triglycerides (TG). Plasma levels of insulin were measured by chemiluminescent microparticle immunoassay using an analyzer (i-2000Abbott Architect ^®^, Chicago, Illinois, USA). HOMA-IR was derived from fasting glucose and insulin levels [(fasting plasma glucose × fasting serum insulin)/22.5]. As HOMA-IR takes into account both insulin and glucose levels, it may be a more complete index than plasma insulin. hsCRP were determined by high-sensitivity ELISA (BioCheck, Inc., Foster City, CA, USA) at the University College Dublin. Large triacylglycerol-rich lipoproteins fraction (TRL) containing chylomicrons and VLDL was removed from plasma by ultracentrifugation performed in a 70Ti fixed-angle rotor (Beckman Instruments, Fullerton, CA, USA) at 30,000 rpm and 4 °C during 30 min at density <1.006 g/mL.

### Statistic analysis

All statistical analyses were made with PASW Statistics software, version 21.0.0. Continuous variables were compared using Student’s “t” and the analysis of variance (ANOVA) depending on the existence of two or more groups in each comparison. When these variables did not follow a normal distribution, the required transformation of the data using for analysis the log10. Data are presented as mean ± standard deviation for continuous variables and as frequencies or percentages for categorical variables. Qualitative variables were compared using Chi square test. To determine the influence of prediabetes in the postprandial metabolism, we used a general linear model of repeated measures of each postprandial parameter with the different groups, blood drawn time as within-subject variable and age, gender, BMI, waist circumference and pharmacological treatments as covariates. We used total area under the curve (AUC) and delta (Δ) AUC of the different postprandial parameters following the trapezoid rule to assess the magnitude of change during postprandial state as in previous works of our group [14]. Bonferroni’s test was used in the post hoc analysis. All analyses were adjusted for potential confounders and p < 0.05 was considered to be significant.

## Results

Baseline demographic and metabolic characteristics according to the patients are presented in Table 1. Age, BMI, waist circumference, HbA1c plasma levels, triglycerides and HOMA-IR were statistically significant among groups (Table 1). We explored the dynamic response according to the diabetic status in the CORDIOPREV population: 57 non diabetic, 364 prediabetic and 581 diabetic.

**Table 1:** Baseline characteristics according to diabetic status

	Diabetic n = 581	Prediabetic n = 364	Non-diabetic n = 57	p value
Age (years old)	60.78 ± 8.67*	58.41 ± 9.28*	54.68 ± 8.39*	0.005
BMI	31.73 ± 4.54*	30.57 ± 4.26*	28.1 ± 4.54*	0.001
Waist circumference (cm)	107.14 ± 11.69*	103.32 ± 19.88*	96.59 ± 10*	0.001
HbA1c (%)	7.19 ± 1.26*	5.96 ± 0.28*	5.39 ± 0.21*	0.001
AST (mg/dL)	24.72 ± 16.67	26.31 ± 13.16	24 ± 6.37	0.219
ALT (mg/dL)	27.96 ± 23.73	28.58 ± 16.52	27.28 ± 13.88	0.136
Fasting triglycerides (mg/dL)	144.88 ± 72.72*	124.76 ± 62.81	105.75 ± 62,22	0.040
CRP (mg/dL)	2.76 ± 2.13	2.08 ± 1.86	1.61 ± 1.46	0.090
Insulin (mU/L)	12.51 ± 13.50	9.19 ± 6.60	6.92 ± 3.87	0.016
Serum ferritin (ng/mL)	97.45 ± 101.14	108.39 ± 100.77	114.58 ± 99.90	0.001
HOMA-IR	5.49 ± 6.49*	2.13 ± 1.60*	1.56 ± 0.94*	0.001
Fasting glucose (mg/dL)	128.53 ± 44.24*	94.11 ± 10.24	81.18 ± 6.7	0.001
Glucose 2 h OGTT (mg/dL)	257.28 ± 108.35*	125.13 ± 38.35	106.11 ± 21.09	0.001
HIRI	2227.52 ± 2631.69*	1170.36 ± 904.67*	768.57 ± 406.98*	0.001
MIRI	0.025 ± 0.016	0.019 ± 0.021	0.022 ± 0.044	0.100

Values are mean ± SD. One way ANOVA

*BMI* body mass index, *HbA1c* glycated hemoglobin, *hs*CRP high sensitivity C-reactive protein, *HOMA-IR* homeostatic model assessment insulin resistance, *OGTT* standard overload glucose tolerance test, *HIRI* hepatic insulin resistance index, *MISI* muscle insulin resistance index

* p < 0.05 posthoc Bonferroni analysis according three subgroups

**Fig. 1:**
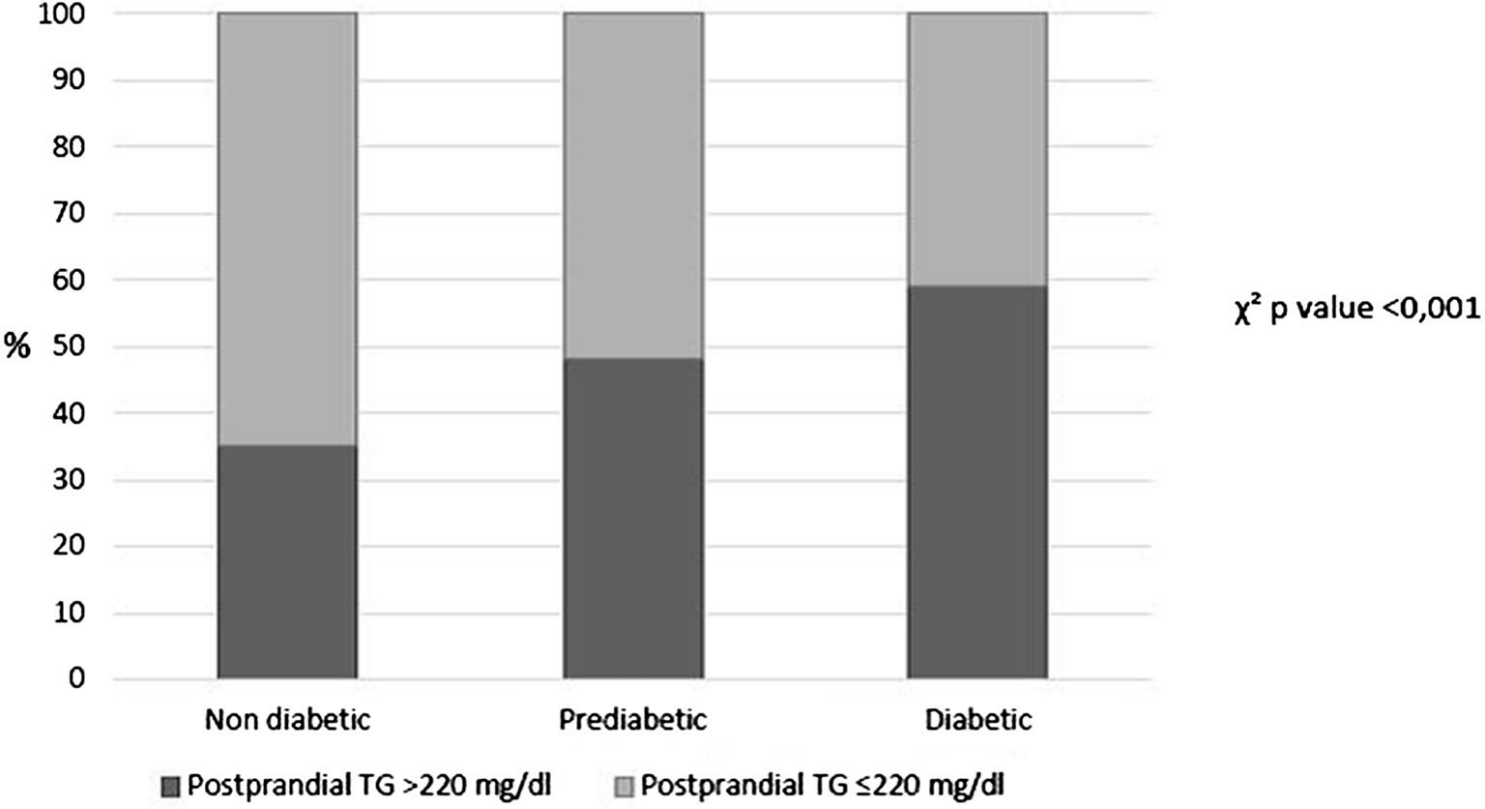
Prevalence of undesirable postprandial triglycerides (TG) in the CORDIOPREV population according to diabetic status: non-diabetic, prediabetic and diabetic subgroups. The *black bars* represent the percentage of patients with postprandial TG concentration at any point >220 mg/dL within each group

We explored the dynamic response during the OFTT in order to identify those subjects with undesirable postprandial TG concentration at any point >2.5 mmol/L or 220 mg/dL. Thus, the prevalence an unsiderable response after the OFTT was 35 % in non-diabetic, 48 % in prediabetic, and 59 % in diabetic subgroup (p < 0.001) (Fig. 1). After the OFTT, diabetic patients showed greater postprandial response of TG (p < 0.001) and large triacylglycerol-rich lipoproteins (TRLs)-TG (p = 0.002) compared with the prediabetic subgroup. Consistently, the AUC-TG and AUC TRLs-TG was significative greater in diabetic patients (p < 0.001 and p = 0.002 respectively) compared with those prediabetic patients (Table 2). Interestingly, prediabetic patients showed higher postprandial response compared with those non-diabetic (p < 0.001). Consequently, non-diabetic patients displayed lower postprandial response of TRLs-TG and AUC-TG, compared with prediabetic and diabetic patients, (p = 0.003 and p < 0.002) (Table 2).

**Table 2:** Postprandial area under the curve (AUC) an incremental (iAUC) of TG and TRLs-TG according to the diabetes status

	Diabetic n = 581	Prediabetic n = 364	Non Diabetic n = 57	p value
AUC-TG	48153.31 ± 21486.26*	42542.64 ± 18250.47*	37612.5 ± 16874.66*	0.001
AUC TRLs-TG	20319.69 ± 16633.45*	17064.00 ± 10771.03*	14970.29 ± 8080.98*	0.001
iAUC-TG	14824.47 ± 10363.01*	13348.15 ± 8737.19	12284.71 ± 9291.00	0.038
iAUC-TRLs-TG	11801.26 ± 8184.73*	10628.53 ± 8179.69	9680.13 ± 6259.98	0.040

Results are plotted as mean ± SE. Variables were compared using ANOVA with age, gender and BMI as covariates. Different letters express statistically significant differences with p value lower than 0.05. Values are mean ± SD. One way ANOVA

*AUC-TG* area under the curve of triglycerides, AUC of the large triacylglycerol-rich lipoproteins (TRLs)-TG, *iAUC-TG* incremental of the area under the curve of triglycerides, *iAUC-TRLs-TG* incremental of the area under the curve of the large triacylglycerol-rich lipoproteins (TRLs)-TG triglycerides

* p < 0.05 posthoc analysis according three subgroups

**Fig. 2:**
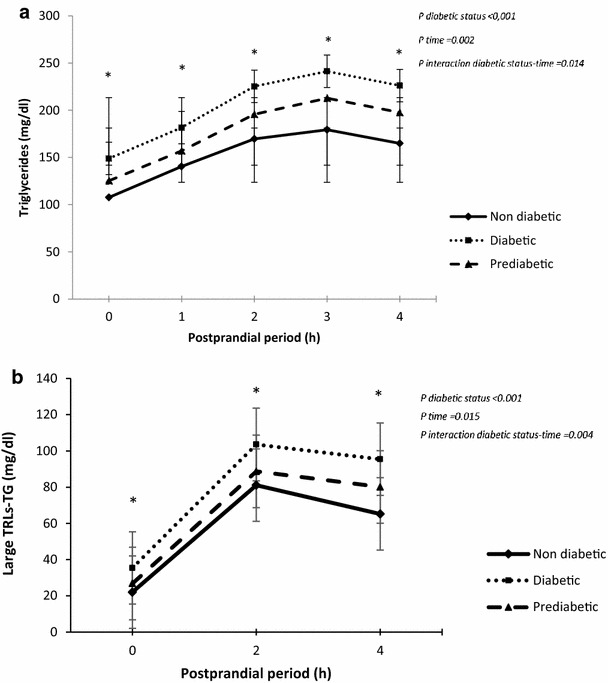
Evolution of (**a**) triglycerides (TG) and (**b**) large triacylglycerol-rich lipoproteins (TRLs)-TG after the oral fat tolerance test, according to the presence of prediabetes, non-diabetes or diabetes state. Results are plotted as mean ± SD. Variables were compared using repeated measured ANOVA, with age, gender and BMI as covariates

In addition, the magnitude of the postprandial response increased progressively in relation to non-diabetic, prediabetic and diabetic state groups (p < 0.001) (Fig. 2a, b). Moreover, AUC-TG and AUC TRLs-TG showed the same effect (p < 0.001 and p < 0.001 respectively) (Table 2). Likewise, diabetic patients compared with prediabetic and non-diabetic subgroups showed higher increase of AUC (iAUC) of TG and iAUC-TRLs-TG (p < 0.001 and p = 0.04 respectively).

**Fig. 3:**
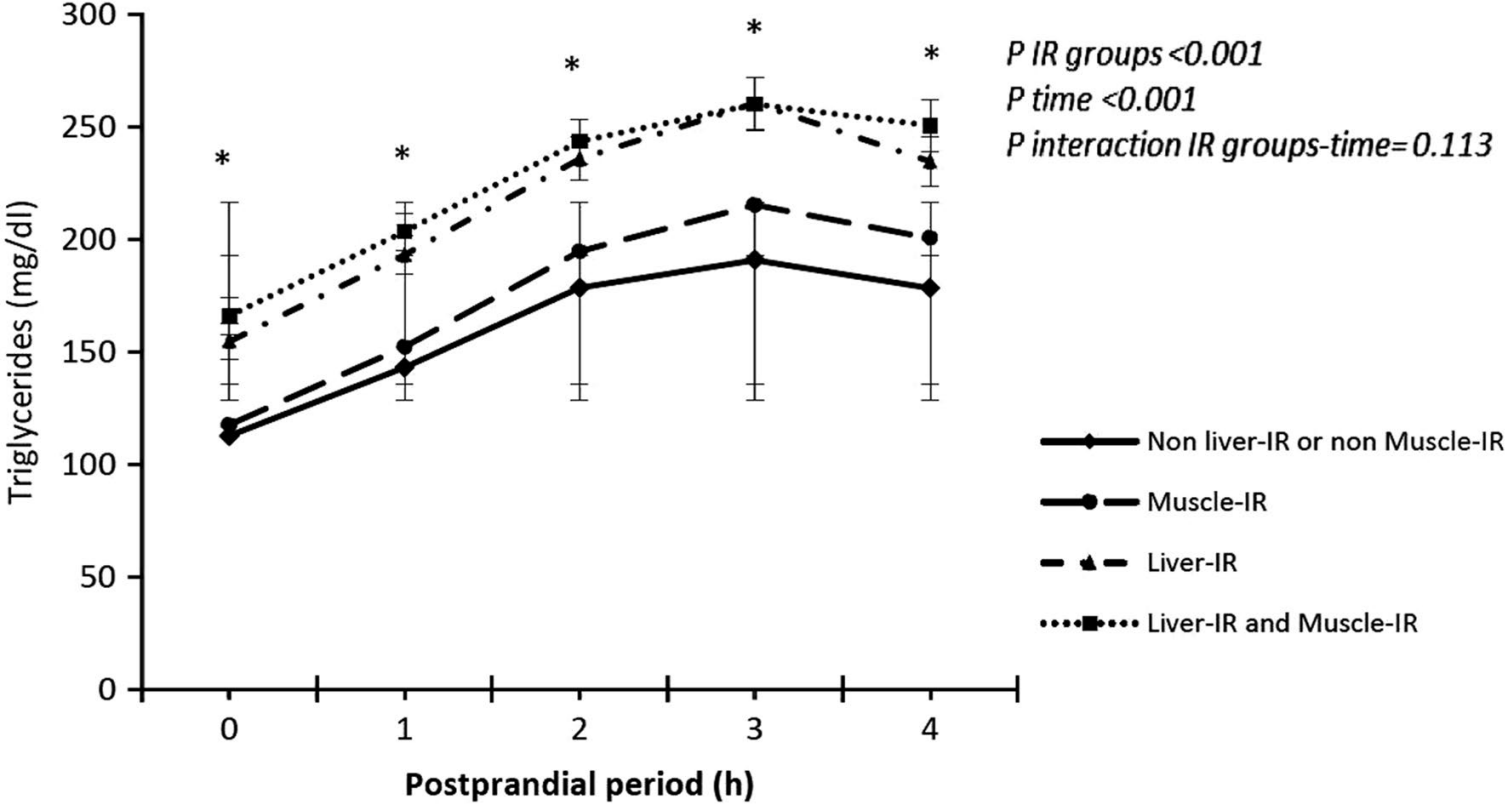
Evolution of triglycerides (TG) after the oral fat tolerance test according to the different basal insulin-resistance groups: muscle-IR and liver-IR, non muscle-IR and non liver-IR, liver-IR and muscle-IR. Results are plotted as mean ± SD. Variables were compared using repeated measured ANOVA, with age, gender and BMI as covariates

**Fig. 4:**
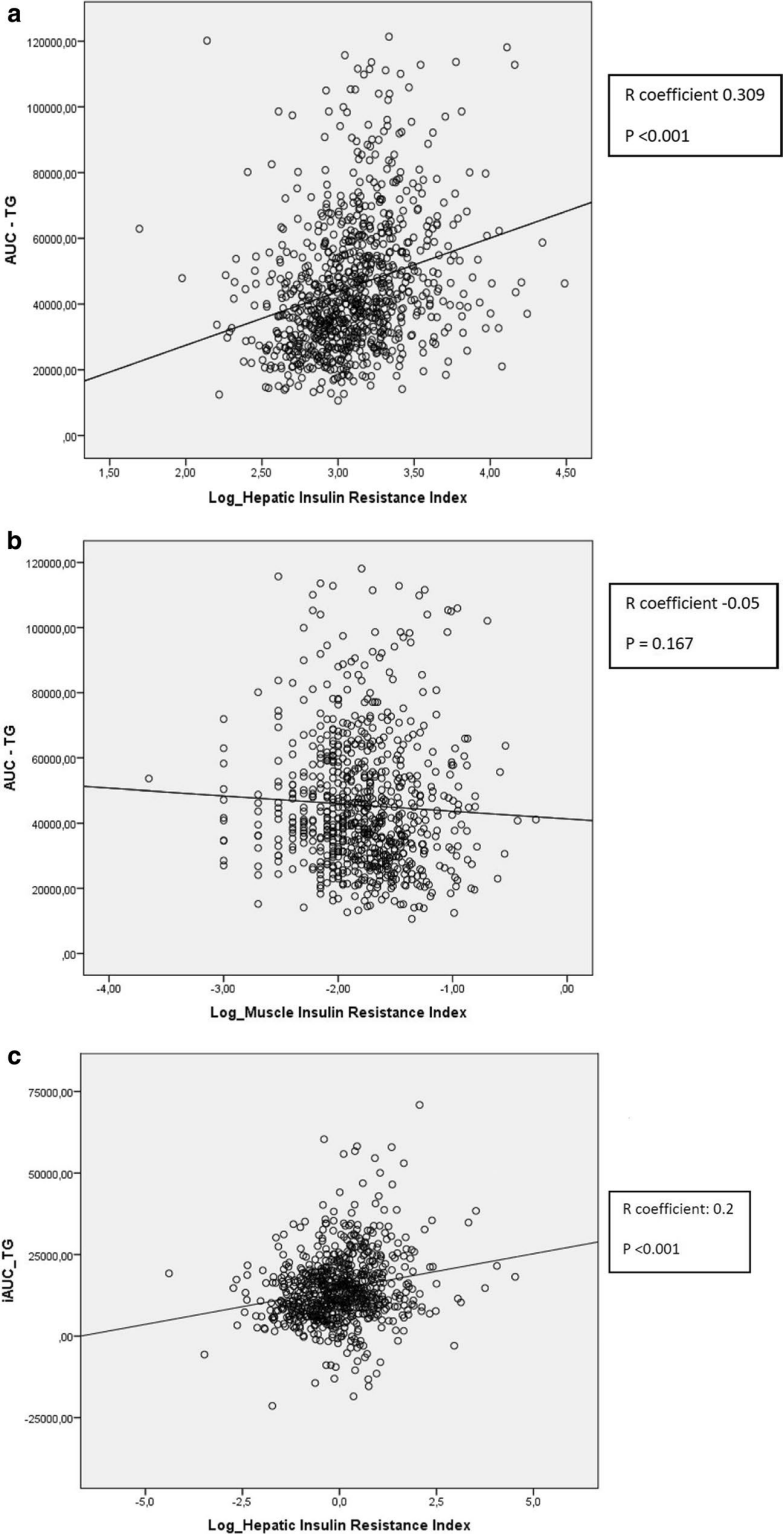
Dispersion diagram and regression line according to AUC-TG and logarithm of HIRI (**a**) and MISI (**b**). Dispersion diagram and regression line according to iAUC-TG and logarithm of HIRI (**c**)

Furthermore, the dynamic response was evaluated in non-diabetic patients and in diabetic patients without pharmacological treatment according to the different groups of baseline insulin resistance: liver-IR, muscle-IR, liver and muscle-IR, non-liver and non-muscle-IR. Patients with liver insulin resistance (liver-IR) showed higher postprandial response of TG compared with those patients with muscle-IR or without any insulin-resistance respectively (p < 0.001). No differences were observed according to the magnitude of postprandial response in group of patients with liver-IR group compared with those patients with liver-IR and muscle-IR (p > 0.05) (Fig. 3). Pearson’s correlation and linear regression were used to associate postprandial response of TG and insulin resistance indices variables: HIRI and MISI. Multiple regression analysis using the AUC-TG as dependent variable showed a significant association with HIRI (R: 0.309; CI 95 % (15327.162–24080.365); p < 0001). It has not been observed association between postprandial response and muscle-IR index. (p > 0.05) (Fig. 4a, b). Similar results were obtained using iAUC-TG as dependent variable. The analysis showed a significant association with HIRI (R: 0.2; IC 95 %: (4437.52–9238.68); p < 0.001). No significant association was observed between postprandial response and MIRI index (R: −0.012; IC 95 %: (−2047.05 to 1439.18); p = 0.732) (Fig. 4c). Finally, we explored the influence of pharmacological treatments (antihypertensives, statins, other hypolipidemic drugs, antiplaquelet, and antidiabetic drugs) on the magnitude of postprandial response and the results did not change.

## Discussion

Our findings support the hypothesis that prediabetic patients showed a lower degree of flexibility by an exaggerated lipoprotein postprandial response, compared with those non-diabetic patients. Moreover, in this large cohort we confirmed previous data indicating that diabetes status is associated with abnormal postprandial lipoprotein metabolism [15, 16]. Thus, the frequency of undesirable response increases progressively according to non-diabetic (35 %), prediabetic (48 %) and diabetic patients (59 %). The postprandial response was higher in patients with liver-IR compared with muscle-IR or without any type of IR. Finally, our results indicate an association between hepatic IR and postprandial-TG response.

Postprandial hypertriglyceridemia is consequence of several factors including genetic variations and medical conditions like obesity, metabolic syndrome and insulin resistance [17, 18]. During the postprandial period, intestinal TRL are the main contributors to the serum lipid level. Hypertriglyceridemia can originate from decreased clearance or increased production of TRL. During lipid absorption, enterocytes produce and secrete chylomicrons and transiently store lipid droplets in the cytosol. The dynamic fluctuation of triglycerides in cytosolic lipid droplets suggests that they contribute to TRL production and may thus control the length and amplitude of the postprandial hypertriglyceridemia [19] Accumulation of TRLs in the postprandial state promotes the retention of remnant particles in the artery wall and for their size these particles cannot cross the endothelium as efficiently as smaller LDL inducing accelerated atherosclerosis [20]. In this regard, previous data have linked the exaggerated postprandial TG response to the incidence of coronary artery disease and stroke [21]. Moreover recent studies have demonstrated that diabetic patients present an exaggerated postprandial TG response after meals [22], and this phenomenon could be translated in a loss phenotypic flexibility and consequently in an increase of cardiovascular disease risk (CVD) [23]. In our large diabetic cohort, our findings consistently confirmed that the diabetic status is associated with an exaggerated postprandial response [15]. Recent data indicate that unlike the situation in the nondiabetic population, in which measurement of postprandial TG levels has been useful in identifying individuals at high CVD, specifically testing for postprandial TG level has shown considerably less promise in T2DM patients. However, the importance for testing postprandial lipemic response in prediabetic patients have not been established yet. According to epidemiological data, up to 70 % of individuals with prediabetes will eventually develop diabetes. Likewise, data from observational studies suggest that prediabetes may also convert back to normoglycaemia [24]. For this reason, we decided to explore the prediabetes status, firstly because it is a reversible state with often asymptomathic period in its early stages [25] and secondly because several trials have demonstrated reductions in the risk of developing diabetes among prediabetics after lifestyle and drug-based interventions [25–27]. In our study, we observed that prediabetic patients displayed higher plasma TG and TRLs-TG postprandial response compared with those non-diabetic patients, suggesting that at this initial stage already have abnormal lipoprotein metabolism. A question arise about what is the main trigger in this process: insulin resistance influencing postprandial lipoprotein response or instead the exaggerated postprandial response favouring the insulin resistance condition. In this context, the physiological link between these both process is not well understood. The hepatocellular TG accumulation may be a direct cause of hepatic insulin resistance. The liver plays a unique role in the regulation of glucose homeostasis by maintaining blood glucose concentration within a normal range. However, impaired insulin action in the liver leads to insulin resistance characterized by impairment in the ability of insulin to inhibit glucose output. Thus, liver insulin resistance which is the reduced sensitivity of the liver to insulin, causes gluconeogenesis and hyperglycemia. As a result of insulin resistance, the adipocyte increased release of free fatty acids (FFA) into the circulation. Increased FFA flux into the liver stimulates hepatic lipogenesis and promotes VLDL-TG overproduction, contributing to the pathogenesis of hypertriglyceridemia in diabetic population [28, 29]. In this regards, recent studies exploring the effect of TRLs on insulin resistance during postprandial lipemia suggest that an exaggerated postprandial lipemia play an important role in the development of diabetes and its associated hepatic insulin resistance, but also in the development of whole body insulin resistance according of these mechanisms [30, 31]. Although the insulin resistance develops simultaneously in multiple organs and it can be defined by different indices, [32] the importance of insulin resistance may differ among the different tissues [33]. In our study we observed that those patients with liver-IR showed higher postprandial TG response compared with those with muscle-IR, and in addition, there was a significant association between postprandial response and hepatic insulin resistance defined by HIRI index. This finding is interesting because suggest that liver-IR appears to be a critical contributor factor of postprandial lipidemia.

From a clinical point of view, to recognize this inflexibility-subgroup of prediabetic patients with an exaggerated postprandial response may be important in terms of early identification of those at greatest risk who should be prioritized for lifestyle intervention according to clinical practice guidelines [34, 35]. In addition, recent studies have shown the possibility of modulating the postprandial response by pharmacological treatments. This point can be the target of future studies in our population [36, 37]. The disadvantage is that the recognition of postprandial hypertriglyceridemia in the clinical setting has been severely hampered by technical difficulties and the lack of established clinical protocols for investigating postprandial lipemia. Although at this point there is no internationally agreed management for postprandial hypertriglyceridemia, a previous consensus has suggested a simple clinical protocol for investigating postprandial TG measurements, and has pointed out cut-offs for undesirable response [TG concentration >2.5 mmol/L (220 mg/dL)] at any time after a OFTT meal. In this context, in this study we explored, the frequency of undesirable postprandial TG response in each subgroup. As expected, diabetic patients commonly showed an undesiderable postprandial TG response and therefore will not benefit diagnostically from an OFTT. However, in the subgroup of prediabetic patients, half of them presented an exaggerated and delayed response and consequently they will benefit diagnostically from an OFTT.

In summary, this study demonstrate that prediabetic patients show a lower metabolic flexibility after external aggression, such as OFTT, compared with nondiabetic patients. The degree of postprandial response increases progressively according to non-diabetic, prediabetic and diabetic state and it is higher in patients with liver insulin-resistance. To identify this subgroup of patients is important to treat more intensively, according to ADA guidelines in order to avoid future cardiometabolic complications.
